# Additions to the genus *Gimesia*: description of *Gimesia alba* sp. nov., *Gimesia algae* sp. nov., *Gimesia aquarii* sp. nov., *Gimesia aquatilis* sp. nov., *Gimesia fumaroli* sp. nov. and *Gimesia panareensis* sp. nov., isolated from aquatic habitats of the Northern Hemisphere

**DOI:** 10.1007/s10482-020-01489-0

**Published:** 2020-11-24

**Authors:** Sandra Wiegand, Mareike Jogler, Christian Boedeker, Anja Heuer, Patrick Rast, Stijn H. Peeters, Mike S. M. Jetten, Anne-Kristin Kaster, Manfred Rohde, Nicolai Kallscheuer, Christian Jogler

**Affiliations:** 1 Institute for Biological Interfaces 5, Karlsruhe Institute of Technology, Eggenstein-Leopoldshafen, Germany; 2 Department of Microbial Interactions, Friedrich-Schiller-University, Jena, Germany; 3 Leibniz Institute DSMZ, Brunswick, Germany; 4 Department of Microbiology, Radboud University, Nijmegen, The Netherlands; 5 Central Facility for Microscopy, Helmholtz Centre for Infection Research, Brunswick, Germany

**Keywords:** *Planctomycetes*, Marine bacteria, *Planctomycetaceae*, Budding, Crateriform structures, Stalk, *Gimesia maris*

## Abstract

Thirteen novel planctomycetal strains were isolated from five different aquatic sampling locations. These comprise the hydrothermal vent system close to Panarea Island (Italy), a biofilm on the surface of kelp at Monterey Bay (CA, USA), sediment and algae on Mallorca Island (Spain) and Helgoland Island (Germany), as well as a seawater aquarium in Braunschweig, Germany. All strains were shown to belong to the genus *Gimesia*. Their genomes cover a size range from 7.22 to 8.29 Mb and have a G+C content between 45.1 and 53.7%. All strains are mesophilic (T_opt_ 26–33 °C) with generation times between 12 and 32 h. Analysis of fatty acids yielded palmitic acid (16:0) and a fatty acid with the equivalent chain length of 15.817 as major compounds. While five of the novel strains belong to the already described species *Gimesia maris* and *Gimesia chilikensis*, the other strains belong to novel species, for which we propose the names *Gimesia alba* (type strain Pan241w^T^ = DSM 100744^T^ = LMG 31345^T^ = CECT 9841^T^ = VKM B-3430^T^), *Gimesia algae* (type strain Pan161^T^ = CECT 30192^T^ = STH00943^T^ = LMG 29130^T^), *Gimesia aquarii* (type strain V144^T^ = DSM 101710^T^ = VKM B-3433^T^), *Gimesia fumaroli* (type strain Enr17^T^ = DSM 100710^T^ = VKM B-3429^T^) and *Gimesia panareensis* (type strain Enr10^T^ = DSM 100416^T^ = LMG 29082^T^). STH numbers refer to the Jena Microbial Resource Collection (JMRC).

**Electronic supplementary material:**

The online version of this article (10.1007/s10482-020-01489-0) contains supplementary material, which is available to authorized users.

## Introduction

The bacterial phylum *Planctomycetes* is part of the PVC superphylum (together with the phyla *Chlamydiae*, *Verrucomicrobia* and other sister phyla) (Wagner and Horn 2006). According to current taxonomy, the phylum *Planctomycetes* features the classes *Planctomycetia*, *Phycisphaerae* and *Candidatus* Brocadiae. Members of the class *Planctomycetia* in particular occur ubiquitously, but predominantly in aquatic environments (Wiegand et al. 2018). They have been found on various algal surfaces (Bengtsson et al. 2012; Bondoso et al. 2014, 2015, 2017; Lage and Bondoso 2014; Vollmers et al. 2017), on which they can dominate biofilms (Bengtsson and Øvreås 2010; Wiegand et al. 2018). Their metabolic ability to degrade complex carbon substrates (Jeske et al. 2013; Lachnit et al. 2013) makes them important players in global carbon cycling (Wiegand et al. 2018). Planctomycetes are suspected to produce small bioactive molecules (Graça et al. 2016; Jeske et al. 2016; Wiegand et al. 2020). Recently, small molecules belonging to the class of *N*-acyl amino acids have been elucidated from Planctomycetes and were shown to be involved in the modulation of biofilm community compositions (Kallscheuer et al. 2020a). This finding might lead to an explanation why Planctomycetes can dominate algal surfaces without being outcompeted, despite their notoriously slow growth (Wiegand et al. 2018).

Members of the class *Planctomycetia* divide by budding, without employing the canonical divisome proteins, including the otherwise universal FtsZ (Jogler et al. 2012; Pilhofer et al. 2008; Wiegand et al. 2020). Most strains seem to have a biphasic lifestyle, alternating between planktonic swimmer cells and sessile mother cells (Wiegand et al. 2020). Their periplasm can be extremely enlarged and compartmentalised (Acehan et al. 2013; Boedeker et al. 2017), conceivably for the digestion of internalised polysaccharides (Boedeker et al. 2017). Interestingly, Planctomycetes were only recently found to possess a peptidoglycan cell wall (Jeske et al. 2015; van Teeseling et al. 2015), as have members of the closely related phylum *Verrucomicrobia* (Rast et al. 2017).

The class *Planctomycetia* has recently been taxonomically restructured and now contains four different orders: *Gemmatales*, *Isosphaerales*, *Pirellulales* and the revised *Planctomycetales* (Dedysh et al. 2020b). The latter taxon harbours only a single family, the *Planctomycetaceae*, which contains the oldest descriptions of planctomycetal bacteria. The name-lending but lost *Planctomyces bekefii* was the first described strain of the phylum (Gimesi 1924) and has recently been re-identified and described (Dedysh et al. 2020a). The first described axenic culture of the family was ‘*Planctomyces maris’* (Bauld and Staley 1976), which was later transferred to *Gimesia maris* (Scheuner et al. 2014). Cells of this species have a long stalk on the non-reproductive pole of the mother cell, whereas daughter cells have a single subpolar flagellum (Scheuner et al. 2014). While these features were also found in the recently described species *Gimesia benthica* (Wang et al. 2020), they were however not identified in another recently described species of the genus, *Gimesia chilikensis* (Kumar et al. 2020). While many Planctomycetes produce carotenoids as pigmenting compounds, probably for protection against UV radiation or oxidative stress (Kallscheuer et al. 2019), the three previously described *Gimesia* species possess a white colony colour (Kumar et al. 2020), indicating the inability to produce such pigments.

In this study, we aimed to broaden the current collection of *Gimesia* strains by the description and detailed comparison of morphological, physiological and phylogenetic properties of thirteen isolates whose genomes have been published before and whose species names have already been suggested based on preliminary analyses (Wiegand et al. 2020). We verify the earlier phylogenetic placement and show that the strains belong to two known species and five hitherto undescribed *Gimesia* species.

## Materials and methods

### Cultivation conditions and isolation

For strain isolation and cultivation, M1H NAG ASW medium was used. For medium preparation 0.25 g peptone (Bacto), 0.25 g yeast extract (Bacto), 2.38 g (4-(2-hydroxyethyl)-1-piperazineethane-sulfonic acid) (HEPES) (10 mM), 250 mL artificial seawater (ASW) and 20 mL Hutner’s basal salt solution were mixed in a final volume of 973 mL double distilled water. The pH was adjusted to 7.5 using 5 M KOH and the solution was autoclaved for 20 min at 121 °C. After cooling, the following solutions were added aseptically: 1 mL of 25% (w/v) glucose, 5 mL vitamin solution, 1 mL trace element solution and 20 mL of a stock solution with 50 g/L *N*-acetyl glucosamine (NAG). ASW contained 46.94 g/L NaCl, 7.84 g/L Na_2_SO_4_, 21.28 g/L MgCl_2_ × 6H_2_O, 2.86 g/L CaCl_2_ × 2H_2_O, 0.384 g/L NaHCO_3_, 1.384 g/L KCl, 0.192 g/L KBr, 0.052 g/L H_3_BO_3_, 0.08 g/L SrCl_2_ × 6H_2_O and 0.006 g/L NaF and was freshly prepared before addition to the base solution. Hutner’s basal salt solution was prepared by first dissolving 10 g nitrilotriacetic acid in 700 mL double distilled water and adjusting the pH to 7.2 using 5 M KOH. Subsequently, the following compounds were added: 29.7 g MgSO_4_ × 7H_2_O, 3.34 g CaCl_2_ × 2H_2_O, 0.01267 g Na_2_MoO_4_ × 2H_2_O, 0.099 g FeSO_4_ × 7H_2_O and 50 mL metal salt solution 44. The solution was filled up to 1 L, sterilised by filtering and stored at 4 °C. Metal salt solution 44 consisted of 250 mg/L Na_2_-EDTA, 1095 mg/L ZnSO_4_ × 7H_2_O, 500 mg/L FeSO_4_ × 7H_2_O, 154 mg/L MnSO_4_ × H_2_O, 39.5 mg/L CuSO_4_ × 5H_2_O, 20.3 mg/L CoCl_2_ × 6H_2_O and 17.7 mg/L Na_2_B_4_O_7_ × 10H_2_O. In the first step, EDTA was dissolved and, if required, a few drops of concentrated H_2_SO_4_ were added to retard precipitation of the heavy metal ions. Metal salt solution 44 was sterilised by filtration and stored at 4 °C. Vitamin solution contained per litre: 10 mg *p*-aminobenzoic acid, 4 mg biotin, 20 mg pyridoxine hydrochloride, 10 mg thiamine hydrochloride, 10 mg Ca-pantothenate, 4 mg folic acid, 10 mg riboflavin, 10 mg nicotinamide and 0.2 mg vitamin B_12_. *p*-Aminobenzoic acid was dissolved first and the solution was sterilised by filtration and stored in the dark at 4 °C. The trace element solution, containing 1.5 g/L Na-nitrilotriacetate, 500 mg/L MnSO_4_ × H_2_O, 100 mg/L FeSO_4_ × 7H_2_O, 100 mg/L Co(NO_3_)_2_ × 6H_2_O, 100 mg/L ZnCl_2_, 50 mg/L NiCl_2_ × 6H_2_O, 50 mg/L H_2_SeO_3_, 10 mg/L CuSO_4_ × 5H_2_O, 10 mg/L AlK(SO_4_)_2_ × 12H_2_O, 10 mg/L H_3_BO_3_, 10 mg/L NaMoO_4_ × 2H_2_O and 10 mg/L Na_2_WO_4_ × 2H_2_O, was sterilised by filtration and stored in the dark at 4 °C.

The sampling location and collected material for isolation of the strains described here are listed in Table S1. Colonies obtained from the initial cultivation step on M1H NAG ASW plates solidified with agar or gellan gum were re-streaked on a new plate and maintained in liquid M1H NAG ASW medium. Initial amplification and sequencing of the 16S rRNA gene was performed as previously described (Rast et al. 2017). This step was included to check whether the strains obtained are members of the phylum *Planctomycetes*.

### Light microscopy

Phase contrast (Phaco) analyses were performed employing a Nikon Eclipse Ti inverted microscope with a Nikon DS-Ri2 camera (blue LED). Specimens were immobilised in MatTek glass bottom dishes (35 mm, No. 1.5) employing a 1% (w/v) agarose cushion (Will et al. 2018). Images were analysed using the Nikon NIS-Elements software (version 4.3). To determine the cell size, at least 100 representative cells were counted manually (Annotations and Measurements, NIS-Elements Imaging Software, Nikon Instruments Europe) or by using the NIS-Elements semi-automated object count tool (smooth: 4×, clean: 4×, fill holes: on, separate: 4×). The Object Count tool enables setting a threshold for the image, automatically measures the binary objects and exports the measured data to a file.

### Electron microscopy

For field emission scanning electron microscopy, bacteria were fixed in 1% (v/v) formaldehyde in HEPES buffer (3 mM HEPES, 0.3 mM CaCl_2_, 0.3 mM MgCl_2_, 2.7 mM sucrose, pH 6.9) for 1 h on ice and washed once employing the same buffer (Rast et al. 2017). Cover slips with a diameter of 12 mm were coated with a poly-l-lysine solution (Sigma-Aldrich) for 10 min, washed in distilled water and air-dried. 50 µL of the fixed bacterial solution was placed on a cover slip and allowed to settle for 10 min. Cover slips were then fixed in 1% (v/v) glutaraldehyde in TE buffer (20 mM TRIS, 1 mM EDTA, pH 6.9) for 5 min at room temperature and subsequently washed twice with TE buffer before dehydrating in a graded series of acetone (10, 30, 50, 70, 90, 100% (v/v)) on ice for 10 min at each concentration. Samples from the 100% acetone step were brought to room temperature before placing them in fresh 100% acetone. Samples were then subjected to critical-point drying with liquid CO_2_ (CPD 300, Leica). Dried samples were covered with a gold/palladium (80/20) film by sputter coating (SCD 500, Bal-Tec) before examination in a field emission scanning electron microscope (Zeiss Merlin) using the Everhart–Thornley HESE2 detector and the inlens SE detector in a 25:75 ratio at an acceleration voltage of 5 kV.

### Physiological analyses

For determination of the temperature optimum for growth, all strains were cultivated in M1H NAG ASW medium at pH 7.5. For determination of the pH optimum for growth, 100 mM HEPES was used instead of 10 mM for cultivations at pH 7.0, 7.5 and 8.0. For cultivation at pH 5.0–6.0 HEPES was replaced by 100 mM 2-(*N*-morpholino)ethanesulfonic acid (MES), whereas 100 mM *N*-cyclohexyl-2-aminoethanesulfonic acid (CHES) served as a buffering agent at pH 9.0–10.0. Cultivations for determination of the pH optimum were performed at 28 °C. For determination of the temperature optimum, all strains were cultivated at pH 8.0 at different temperatures ranging from 10 to 40 °C.

### Cellular fatty acid analysis

Biomass of all tested strains was obtained from liquid cultures grown in M1H NAG ASW medium under optimal growth conditions until cells reached the stationary phase. 30 mg of lyophilised biomass was analysed by the Identification Service of the German Collection of Microorganisms and Cell Cultures (DSMZ) according to standard protocols (Kämpfer and Kroppenstedt 1996; Kuykendall et al. 1988; Miller 1982).

### Phylogenetic analyses

The genomes of all strains were published previously (Wiegand et al. 2020) and are available from RefSeq under accession numbers NZ_CP036269.1, NZ_CP036343.1, NZ_CP037920.1, NZ_CP037422.1, NZ_CP036266.1, NZ_CP036342.1, NZ_CP036347.1, NZ_CP037452.1, NZ_CP036353.1, NZ_CP036341.1, NZ_CP037421.1, NZ_CP036277.1 and NZ_CP036317.1. The GenBank accession numbers of the respective 16S rRNA genes are MK554516, MK554515, MK554556, MK554536, MK554525, MK554514, MK554557, MK554524, MK559968, MK554512, MK554508, MK559981 and MK554531. 16S rRNA gene sequence-based phylogeny was computed for the strains described here and the type strains of reference species. The analysis also included strains described in the last year (Boersma et al. 2019; Dedysh et al. 2020a; Kallscheuer et al. 2020b; Kohn et al. 2016, 2019, 2020; Kumar et al. 2020; Peeters et al. 2020; Rivas-Marin et al. 2020a, b; Vitorino et al. 2020) and strains recently published, but not yet described (Wiegand et al. 2020). The 16S rRNA gene sequences were aligned with SINA (Pruesse et al. 2012). The phylogenetic analysis was performed employing a maximum likelihood approach with 1000 bootstraps, the nucleotide substitution model GTR, gamma distribution and estimation of proportion of invariable sites (GTRGAMMAI option) (Stamatakis 2014). Three 16S rRNA genes of bacterial strains from the PVC superphylum (outside of the phylum *Planctomycetes*) served as outgroup. The *rpoB* nucleotide sequences (encoding the RNA polymerase β-subunit) were taken from publicly available genome annotations and the sequence identities were determined as previously described (Bondoso et al. 2013) with Clustal Omega (Sievers et al. 2011). Alignment and matrix calculation were performed upon extracting only those parts of the sequence that would have been sequenced with the primer set described by Bondoso et al. (2013).

The average nucleotide identity (ANI) was calculated using OrthoANI (Lee et al. 2016). The average amino acid identity (AAI) was obtained using the aai.rb script of the enveomics collection (Rodriguez-R and Konstantinidis 2016) and the percentage of conserved proteins (POCP) was calculated as described before (Qin et al. 2014).

For the multilocus sequence analysis (MLSA), the unique single-copy core genome of all analysed genomes was determined with proteinortho5 (Lechner et al. 2011) with the ‘selfblast’ option enabled. The protein sequences of the resulting orthologous groups were aligned using MUSCLE v.3.8.31 (Edgar 2004). After clipping, partially aligned *C*- and *N*-terminal regions and poorly aligned internal regions were filtered using Gblocks (Castresana 2000). The final alignment of 900 ubiquitous genes with a combined length of 440,140 conserved amino acid residues was concatenated and clustered using FastTree (Price et al. 2010). The outgroup consisted of three genomes from strains of the family *Pirellulaceae*.

### Genomic analysis

The genome-based analysis of enzymes participating in primary metabolism was performed by examining locally computed InterProScan (Mitchell et al. 2019) results cross-referenced with information from the UniProt (UniProt 2019) database and BLASTp results of ‘typical’ protein sequences. The local alignment of the genomes was computed with BLASTn and visualised with Easyfig (Sullivan et al. 2011). The analysis of the pan genomes was performed with anvi’o (Eren et al. 2015), following the pangenomics workflow (Delmont and Eren 2018). The origin of genes of primary metabolism was tested by querying the BLAST nr database with the gained genes from the novel strains (maximal target sequences: 250), concatenation and deduplication of the results, subsequent alignment by MUSCLE (Edgar 2004), tree generation with FastTree 2 (Price et al. 2010) and visualisation with iTOL v4 (Letunic and Bork 2019).

## Results and discussion

### Isolation of the strains

Strains CA11, Mal35, Pan161^T^, Pan241w^T^, Enr17^T^, V202, V144^T^, MalM14, HG66A1, V6, Pan110, Enr10^T^ and Pan153 were previously reported (Wiegand et al. 2020) as novel isolates gained during a large diversity-driven cultivation approach targeting the phylum *Planctomycetes*. The 13 strains were isolated from sediments or biofilms from five different aquatic sampling locations: the Monterey Bay kelp forest (CA11), the hydrothermal vent system offshore of Panarea Island (Enr10^T^, Enr17^T^, Pan 110, Pan 153, Pan161^T^, Pan241w^T^), a public beach at Mallorca Island (Mal35, MalM14), rocky tideland at Helgoland Island (HG66A1) and a seawater ornamental fish tank (V6, V144^T^, V202). More details are provided in Table S1.

### Phylogenetic inference

**Fig. 1:**
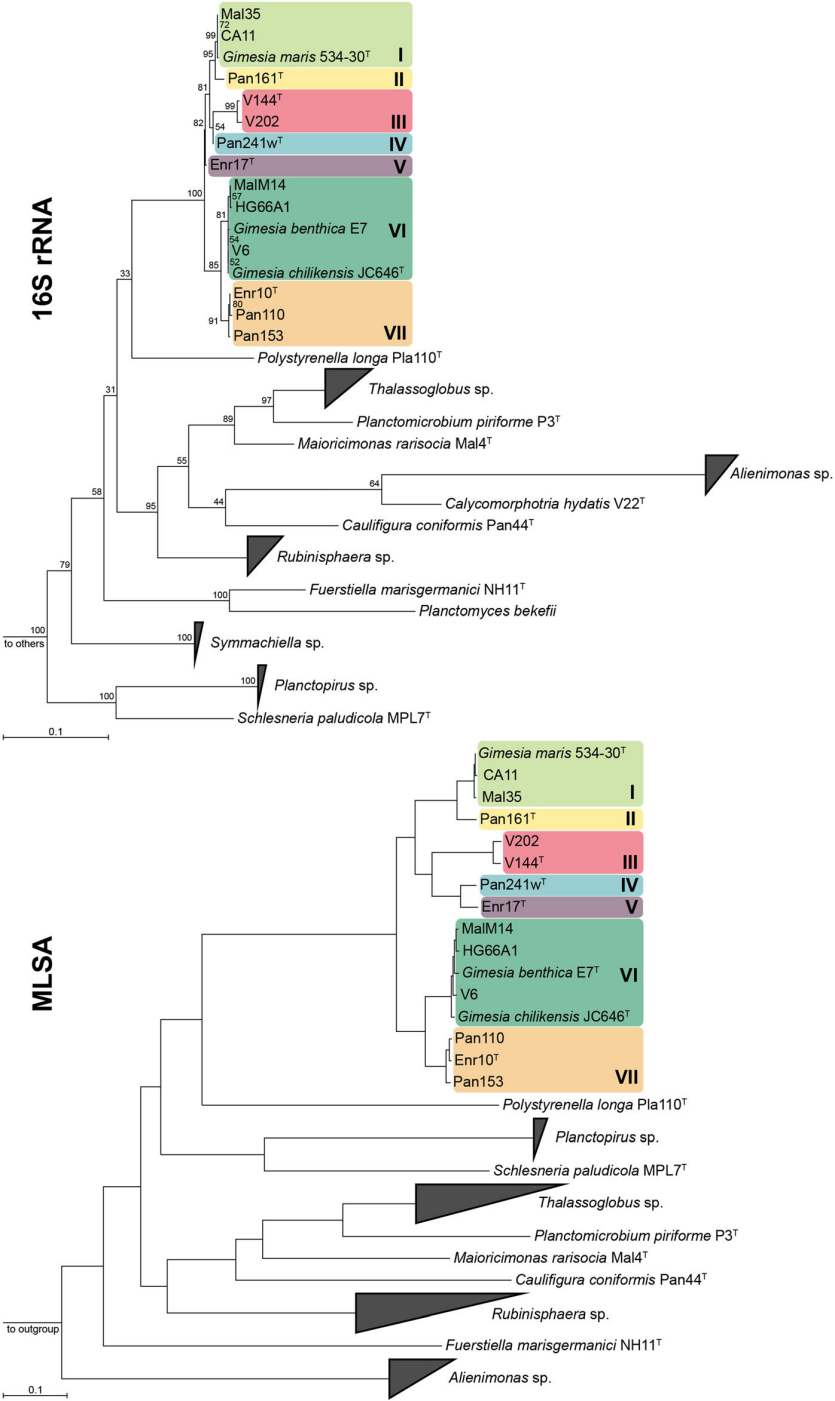
Phylogeny of the family *Planctomycetaceae* based on 16S rRNA gene sequences and whole genome-based multilocus sequence analysis (MLSA). The recently described species of the genera *Thalassoglobus*, *Maioricimonas, Polystyrenella, Alienimonas* and *Caulifigura* are included. The novel strains, the type species of the genus *Gimesia*, *G. maris 534*-*30*^T^, as well as *G. chilikensis* JC6446^T^ and *G. benthica* E7 are colour-coded depending on their group affiliation: (I) light green, (II) yellow, (III) red, (IV) blue, (V) purple, (VI) dark green and (VII) orange. For the 16S rRNA analysis, maximum likelihood estimation-gained bootstrap values after 1000 re-samplings are given at the nodes (in %). The outgroup consists of three 16S rRNA genes from the PVC superphylum. Members of other planctomycetal families are not shown. For the MLSA-based phylogeny, reliability estimators based on Shimodaira–Hasegawa testing were determined but are not shown as they were always 1. The outgroup contains three genomes from the family *Pirellulaceae*. (Color figure online)

When reviewing 16S rRNA gene sequence-, as well as MLSA-based phylogeny, it was evident that all strains are close neighbours of *G. maris* 534-30^T^ (Bauld and Staley 1976; Scheuner et al. 2014), the type strain of the type species of the genus *Gimesia*, and the recently described *G. chilikensis* JC646^T^ (Kumar et al. 2020) and *G. benthica* E7 (Wang et al. 2020) (Fig. 1). By closer examination of the phylogenetic trees, the strains can be subdivided into seven groups: (I) *G. maris* 534-30^T^, CA11, Mal35; (II) Pan161^T^; (III) V202, V144^T^; (IV) Pan241w^T^; (V) Enr17^T^; (VI) *G. chilikensis* JC646^T^, *G. benthica* E7, MalM14, HG66A1, V6; and (VII) Pan110, Enr10^T^ and Pan153. Interestingly, the geographic origin of the strains is not closely reflected in these groups; while groups V and VII only comprise strains from one location, in groups I and VI each strain is derived from a different sampling spot.

**Fig. 2:**
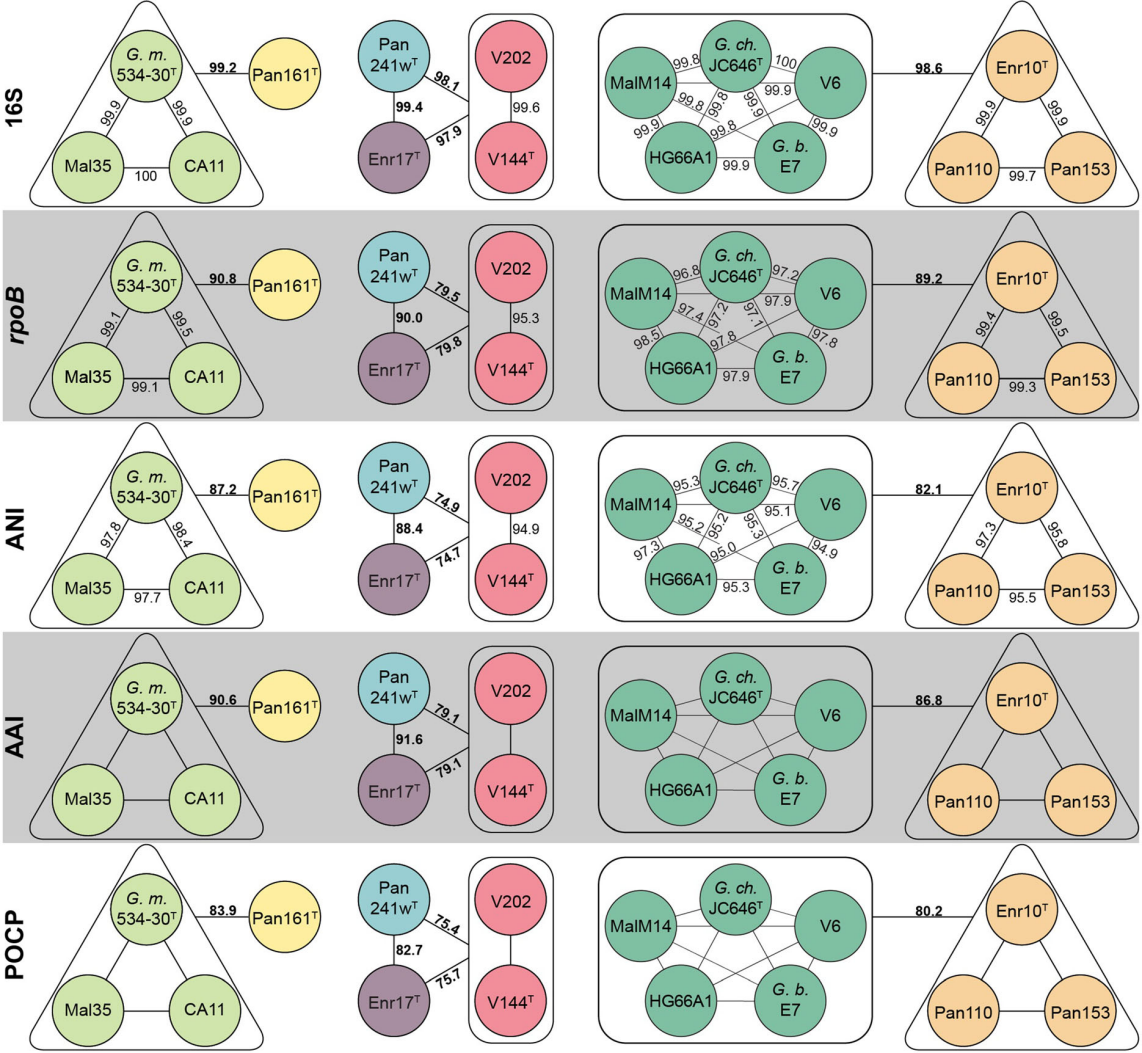
Phylogenetic markers used for species and genus attribution. Strains suspected to belong to the same species (groups I to VII) have the same colour: (I) light green, (II) yellow, (III) red, (IV) blue, (V) purple, (VI) dark green and (VII) orange. Values (always in %) determined for average nucleotide identity (ANI), *rpoB* gene identity and 16S rRNA gene sequence identity can be used to delineate species. They are given in black within groups and in black and bold between groups, where groups might be suspected to belong together. (Minimal) identity values between clearly resolved groups are not given in the figure but can be found in Table S2. Amino acid identity (AAI) and percentage of conserved proteins (POCP) values are only shown between groups, as these values are used to determine genus affiliation. All values are given in percent. *G. m.*: *Gimesia maris*, *G. ch.*: *Gimesia chilikensis*, *G. b.*: *Gimesia benthica*. (Color figure online)

The assessment of the seven groups all belonging to the genus *Gimesia* is confirmed by average amino identities (AAI) and the percentage of conserved proteins (POCP): all values are notably above the genus thresholds of 60–80% (Luo et al. 2014) and 50% (Qin et al. 2014), respectively (Fig. 2 and Table S2).

When applying the proposed ANI threshold of 95% for delineation of species (Kim et al. 2014), the seven introduced groups each form a separate species. While all determined values within the groups are higher than 95%, all values between the groups are below 95% (Fig. 2 and Table S2). The same picture emerges for the comparison of *rpoB* gene identities. The species threshold for this marker was proposed to be > 95.5% (Bondoso et al. 2013). With the exception of the value within group III (95.3%)—which is very close to the proposed threshold—all defined groups meet this criterion in terms of forming the same or different species (Fig. 2 and Table S2). For 16S rRNA gene sequence identities, the general relation patterns of the groups can also be confirmed (Fig. 2 and Table S2). However, the well-established species threshold of 98.7% (Yarza et al. 2014) does not seem to be applicable for the novel strains as the identity values found appear to be higher than what would be expected from the ANI and *rpoB* gene sequence identities. However, this is a phenomenon known for the family *Planctomycetaceae*: e.g. novel species in the genus *Planctopirus* were found to reach 16S rRNA sequence identity values of 99.7% and 99.9% despite ANI values < 95% (Kohn et al. 2020). Therefore, we propose to interpret 16S rRNA gene sequence identity with caution and instead rely more strongly on ANI and *rpoB* gene sequence identity values for the classification within this taxonomic group.

Notably, our analysis shows that the two described species *G. chilikensis* (Kumar et al. 2020) and *G. benthica* (Wang et al. 2020) do not seem to belong to different species by the here applied measures. We must assume that this inconsistency derives from the fact that *G. benthica* was described only a few weeks after *G. chilikensis*—and therefore each of these studies only compared the new strain to *G. maris*, rather than to each other. However, the rules of priority mean that *G. chilikensis* JC646^T^ serves as type strain of the species represented by group VI as it was described first.

### Morphology and physiology

**Fig. 3:**
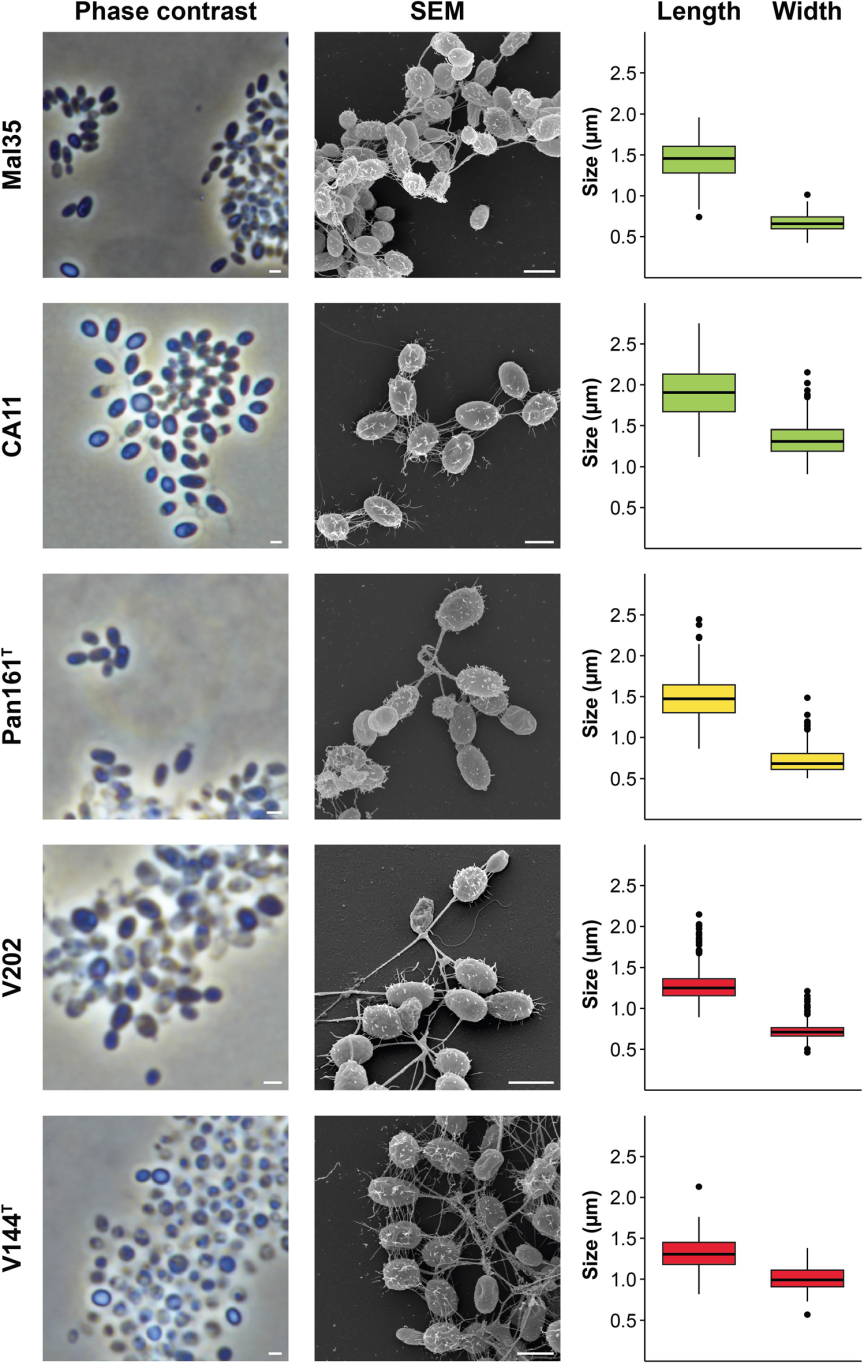
Microscopy images and cell size plot of strains of groups I (green), II (yellow) and III (red). The figure shows phase contrast as well as scanning electron microscopy (SEM) micrographs. The scale bar is 1 µm. For determination of the cell sizes at least 100 representative cells were counted manually or by using a semi-automated object count tool. Whiskers of box plots represent 1.5× interquartile range. (Color figure online)

**Fig. 4:**
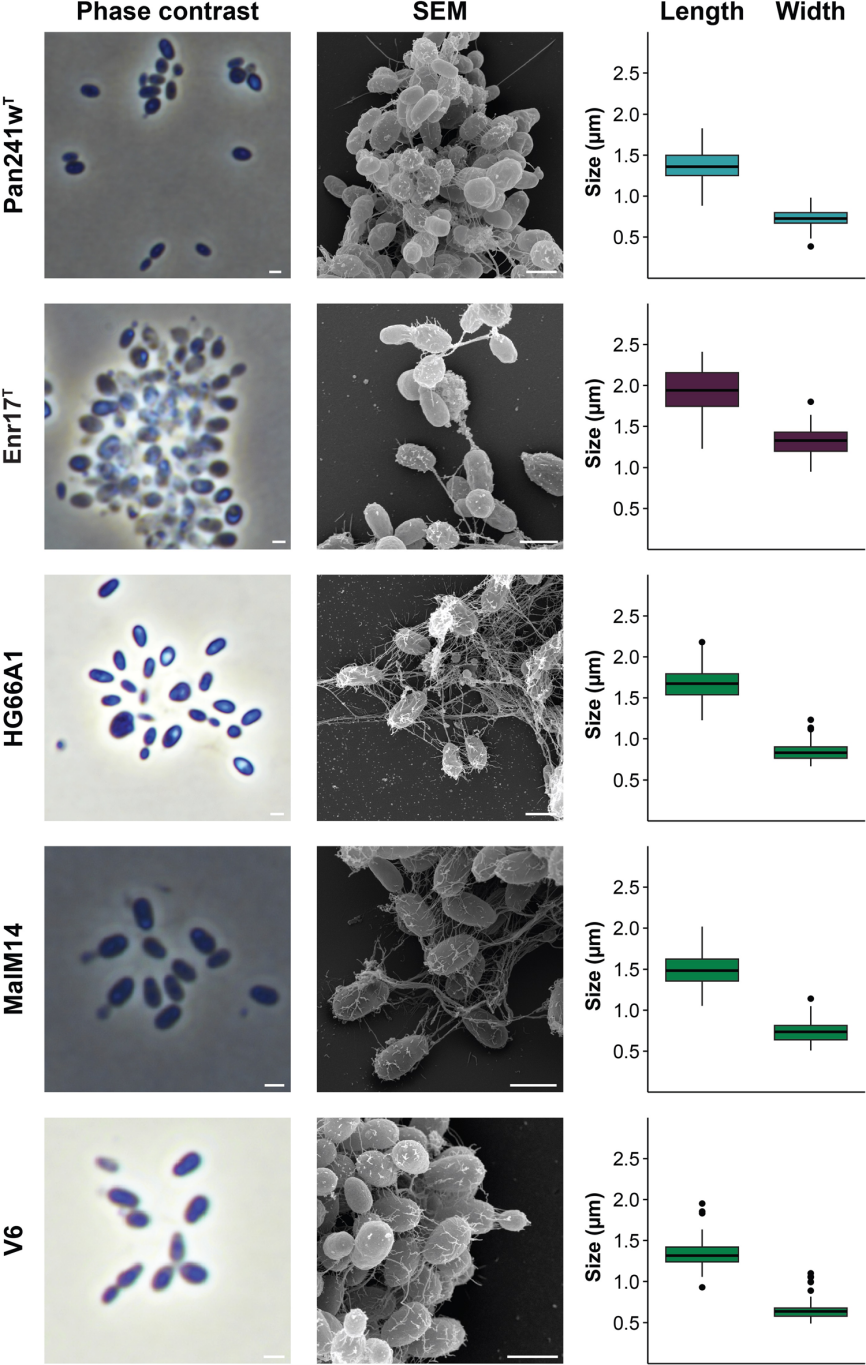
Microscopy images and cell size plot of strains of groups IV (blue), V (purple) and VI (green). The figure shows phase contrast as well as scanning electron microscopy (SEM) micrographs. The scale bar is 1 µm. For determination of the cell sizes at least 100 representative cells were counted manually or by using a semi-automated object count tool. Whiskers of box plots represent 1.5× interquartile range. (Color figure online)

**Fig. 5:**
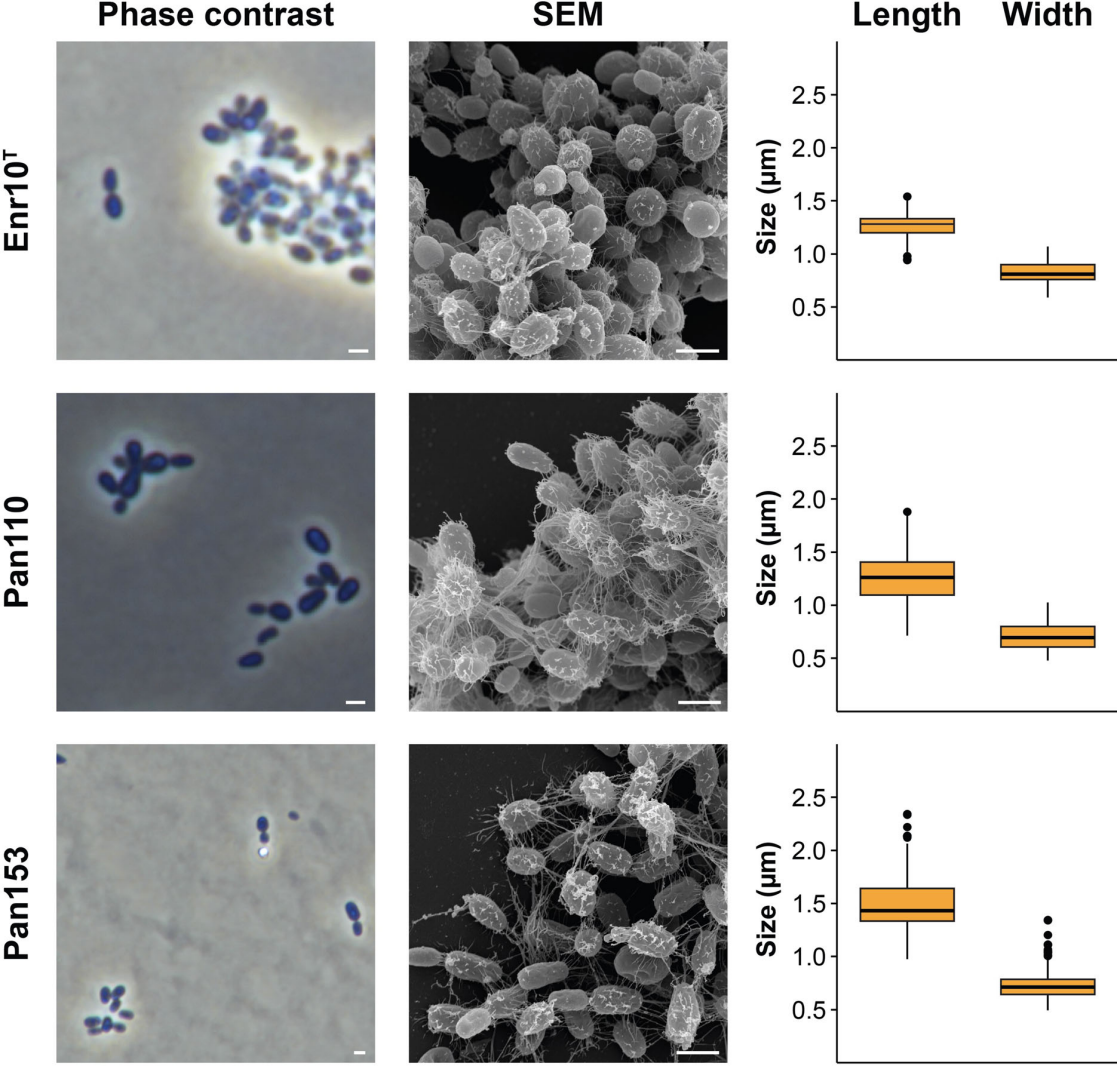
Microscopy images and cell size plot of strains of group VII. The figure shows phase contrast as well as scanning electron microscopy (SEM) micrographs. The scale bar is 1 µm. For determination of the cell sizes at least 100 representative cells were counted manually or by using a semi-automated object count tool. Whiskers of box plots represent 1.5× interquartile range. (Color figure online)

Cell morphology and cell size of strains CA11, Mal35, Pan161^T^, Pan241w^T^, Enr17^T^, V202, V144^T^, MalM14, HG66A1, V6, Pan110, Enr10^T^ and Pan153 were determined by observation using light microscopy and scanning electron microscopy (SEM) during exponential growth (Figs. 3, 4, 5). All strains form ovoid cells resembling the shape of short-grain rice. The cell sizes are given in Figs. 3, 4, 5 and Table S3. On average, they range from 1.3 to 1.9 µm in length and 0.7–1.4 µm in width. All strains divide by budding, with the released buds having the same shape as the mother cells. For all strains with appropriate early exponential phase data, the dimorphic life cycle known from *G. maris* 534-30^T^ (Bauld and Staley 1976) could also be observed (strains V202, HG66A1, Enr17^T^, CA11 and Pan153). All strains form rosettes and larger loose aggregates connected by a stalk formed on the non-budding pole, with fibres covering the entire cell surface. All strains possess flagella at some point of their life cycle and carry crateriform structures as typical for Planctomycetes (Wiegand et al. 2018). All strains grow aerobically. The colonies of all strains are white- to cream-coloured; with the only exceptions being strains V202 and V144^T^ (group III) which form orange colonies. Fatty acid analysis revealed palmitic acid (C_16:0_) and a fatty acid with an equivalent chain length of 15.817 (C_16:1_*ω7c* or C_15_ *iso* 2-OH or C_16:1_*ω6c*) to be major components of all strains, accounting for circa 80% of the total fatty acids (Table 1). The strains are able to grow over pH ranges of 5–10, with the optima being between 6.5 and 8.5 (Table 2, Fig. S1). While most strains are capable of growth over a broad spectrum within this range, strain V202 only grew at pH 7–7.5. Growth for the 13 examined strains could be observed at temperatures between 10 and 37 °C with optima between 26 and 33 °C (Table 2, Fig. S2). Strains belonging to group VI seem to have the highest temperature optima under the tested conditions, while strain Pan161^T^ (group II) had the lowest. The maximal growth rates in M1H NAG ASW medium at the optimal temperature were found to be between 0.022 and 0.057 h^−1^ which corresponds to doubling times between 12 and 32 h (Table 2). Strains of group III were distinctly the slowest-growing strains under the tested conditions.

**Table 1:** Cellular fatty acid contents (%) of the novel strains

	Mal35	CA11	Pan161^T^	Pan241w^T^	Enr17^T^	V144^T^	V202	HG66A1	MalM14	V6	Enr10^T^	Pan110	Pan153
C_12:0_ 3-OH	0.53	0.41	0.62	0.7	0.57	0.51	0.43	0.14	0.31	0.3	0.43	0.35	0.35
C_14:0_	1.22	0.39	0.4	0.18	0.3	0.25	0.17	0.16	0.1	0	0.17	0	0.1
C_14:1_*ω5c*	0.09	0	0	0	0	0	0	0	0	0	0	0	0
C_15:1_*ω6c*	0.88	0.88	0.13	0.37	1.04	0	0	0.26	0.42	0.29	0.45	0.16	0.29
C_15:0_	1.2	1.9	0.22	1.18	3.02	0.16	0.22	1.35	0.76	0.88	0.5	0.55	0.62
ECL 15.488^a^	0.19	0.47	0	0.28	0.26	0.21	0	0	0.28	0	0.27	0.27	0.2
C_16:0_ *iso*	0	0.22	0	0.15	0	0	0	0.22	0.13	0.12	0	0	0
ECL 15.817^b^	51.73	38.56	55.06	39.54	42.09	45.05	43.84	38.8	43.47	44.17	45.81	43.45	44.63
C_16:1_*ω5c*	0.68	0.42	0.43	0.21	0.23	0.37	0.18	0.14	0.15	0.14	0.14	0	0.17
C_16:0_	28.84	31.39	27.14	32.6	32.08	37.38	35.75	34.66	37.26	31.6	31.54	31.89	32.35
C_16:0_ 10-methyl	0.56	7.11	0.6	1.52	2.02	2.09	1.05	4.54	0.4	0.27	0.17	1.12	1.83
C_17:0_ *iso*	0	0	0	0	0	0	0	0.46	0	0	0	0	0
C_17:1_*ω8c*	1.28	1.38	0.9	1.06	1.9	0.19	0.36	1.17	1.61	2.43	2.13	1.53	1.39
C_17:1_*ω6c*	0.84	1.47	0.43	1.24	2.18	0.22	0.22	0.95	0.91	1.1	0.75	0.8	0.89
C_17:0_	1.05	2.65	0.25	2.17	3.32	0.27	0.29	2.19	2.03	1.34	1.41	1.16	1.17
C_17:0_ 10-methyl	0	0.33	0	0	0	0	0	0	0	0	0	0	0
C_18:1_*ω9c*	3.08	3.81	4.27	4.51	2.62	3.24	4.94	3.54	3.55	3.7	4.38	3.8	3.03
C_18:1_*ω7c*	6.36	6.47	8.84	11.8	7.51	8.15	11.08	8.47	6.32	11.29	8.32	11.61	9.28
C_18:0_	1.3	1.94	0.54	1.74	0.85	1.39	0.75	2.08	1.83	0.86	1.01	0.91	0.91
C_18:1_*ω*7c 11-methyl	0	0	0	0	0	0	0.29	0.08	0	0	0.23	0	0
C_17:0_ *iso* 3-OH	0	0	0	0	0	0	0	0.1	0	0	0	0	0
C_19:0_ *anteiso*	0	0	0	0	0	0	0	0	0	0	0	0	0
C_20:1_*ω9c*	0	0	0	0.21	0	0	0	0	0	0	0	0	0
C_20:1_*ω7c*	0.18	0.23	0.17	0.55	0	0.5	0.42	0.69	0.47	1.52	2.32	2.39	2.78

^a^No clear result: C_14:0_ 3-OH or C_16:1_ *iso* I

^b^No clear result: c_16:1_*ω7c* or C_15_ *iso* 2-OH or C_16:1_*ω6c*

**Table 2:** pH and temperature optima for growth and maximal growth rates for the novel strains

	pH	pH_opt_	T (°C)	T_opt_ (°C)	Growth rate at T_opt_ (h^−1^)	Generation time at T_opt_ (h)
Mal35	6.0–9.0	7.0	10–33	27	0.032	22
CA11	5.0–10.0	7.5	10–33	30	0.046	15
Pan161^T^	5.5–10.0	8.5	10–30	26	0.028	25
Pan241w^T^	6.0–10.0	7.5	15–33	30	0.050	14
Enr17^T^	6.0–9.5	7.0	10–30	27	0.046	15
V144^T^	6.5–9.5	8.0	15–30	27	0.022	32
V202	7.0–7.5	7.5	15–30	27	0.024	29
HG66A1	5.0–10.0	6.5	10–36	33	0.057	12
MalM14	6.0–8.0	7.0	15–33	30	0.037	19
V6	5.0–9.5	7.5	17–33	33	0.037	19
Enr10^T^	5.0–10.0	7.5	15–37	32	n.d.	n.d.
Pan110	5.0–10.0	7.5	16–36	31	0.039	18
Pan153	5.5–9.0	8.5	10–36	30	0.048	14

*n.d.* not determined

Genome analysis indicated that all the novel strains carry genes to allow the conversion of glucose to pyruvate via the Embden–Meyerhof–Parnas and the Entner–Doudoroff pathway. They all seem to be capable of performing gluconeogenesis. The pentose phosphate pathway and the citric acid cycle are fully functional according to presence of respective genes coding for the enzymes involved; however, the glyoxylate shunt is probably absent (Table S4). This is in accordance with the described strains *G. maris* and *G. chilikensis*. However, there are some features that allow the different groups to be distinguished. While all members of groups III to VII have a heterodimeric transketolase encoded by two adjacent genes, strains from group I and II have one longer gene coding for the transketolase carrying all necessary domains in a single polypeptide chain (Table S4).

Moreover, members of groups I and II carry glucose-6-phosphate isomerase (*pgi*) genes that are less similar to *pgi* genes of other *Planctomycetaceae*, but seem more related to *pgi* of some *Acidobacteria*, *Proteobacteria* and *Cand.* Rokubacteria. Differentiation between group I and II can be made as strain Pan161^T^, the only member of group II, is the only analysed strain that has multiple paralogs of several genes of primary metabolism (e.g. glucose-6-phosphate isomerase, phosphoenolpyruvate synthase, transketolase and transaldolase) (Table S4).

### Genomic features

The genomes of all strains (Table 3), including the type strain *G. maris* 834-30^T^ are completely closed and were recently published (Wiegand et al. 2020). The genome sizes of the strains are between 7.22 and 8.29 Mb, with members of group III having the smallest genomes. The G+C content of the DNA varies between 45.1 and 53.7%, but is highly conserved within the respective groups. While members of group III each have a low G+C content of 45.1%, members of group VII have the highest G+C content of 53.3–53.7%. The number of proteins per Mb and the number of hypothetical proteins is relatively stable for all strains. Most strains have 63–67 tRNAs, with the exception of strains of group II, which carry 74 and 76 tRNA genes. Interestingly, while all novel strains have one copy of the 23S rRNA gene and the 5S rRNA gene, ten strains have a duplicated 16S rRNA gene.

**Table 3:** Genomic features of the novel strains and the previously described strains *G. maris* 534-30^T^, *G. chilikensis* JC646^T^ and *G. benthica* E7

	Genome size (Mb)	G+C content (%)	Genes	Genes per Mb	Protein-coding genes	Protein-coding genes per Mb	Hypothetical proteins	Coding density	Ribosomal RNA genes 16S–23S–5S	tRNAs	Giant genes	Transposable elements
*G. maris*	7.82	50.4	6064	775	5994	766	3877	86.9	2–1–1	66	8	35
CA11	7.63	50.3	5969	782	5900	773	3792	86.9	2–1–1	65	7	63
Mal35	7.53	50.4	5864	779	5796	770	3728	86.5	1–1–1	64	7	24
Pan161^T^	7.93	50.2	6274	791	6206	783	4001	86.5	2–1–1	64	6	57
Pan241w^T^	7.77	49.6	6113	787	6042	778	3958	87.0	2–1–1	66	7	19
Enr17^T^	7.70	49.5	6146	798	6078	789	3982	87.4	2–1–1	63	7	44
V144^T^	7.40	45.1	5838	789	5759	778	3709	86.6	2–1–1	76	4	1
V202	7.22	45.1	5648	782	5571	772	3542	86.5	1–1–1	74	5	4
*G. chilikensis*	7.65 (7.65)	53.2 (53.2)	6011	786	5941	777	3850	87.5	1–1–1 (3)	66 (67)	4	8
*G. benthica*	8.03 (8.03)	52.7 (52.8)	6472	806	6404	798	4174	86.8	2–1–0	63	3	52
HG66A1	8.29	52.8	6548	790	6480	782	4318	87.2	2–1–1	63	6	21
MalM14	7.80	53.2	6079	779	6012	771	3898	87.2	1–1–1	64	6	14
V6	8.10	52.9	6414	792	6343	783	4181	86.8	1–1–1	68	5	17
Enr10^T^	7.83	53.7	6145	785	6077	776	3952	87.2	2–1–1	63	3	68
Pan110	7.98	53.6	6227	780	6158	772	3979	87.3	2–1–1	65	6	81
Pan153	8.26	53.3	6409	776	6337	767	4144	87.5	2–1–1	67	10	78

The values for *G. chilikensis* were calculated in the same manner as those for the other strains to ensure comparability with Prokka v1.14 (Seemann 2014). In cases where values were given in the original strain descriptions (Kumar et al. 2020; Wang et al. 2020), they are shown in parentheses. Deviation between the data is due to the usage of different prediction algorithms. Giant genes are defined as genes with a length of > 15 kb (coding for proteins > 5000 amino acids)

**Fig. 6:**
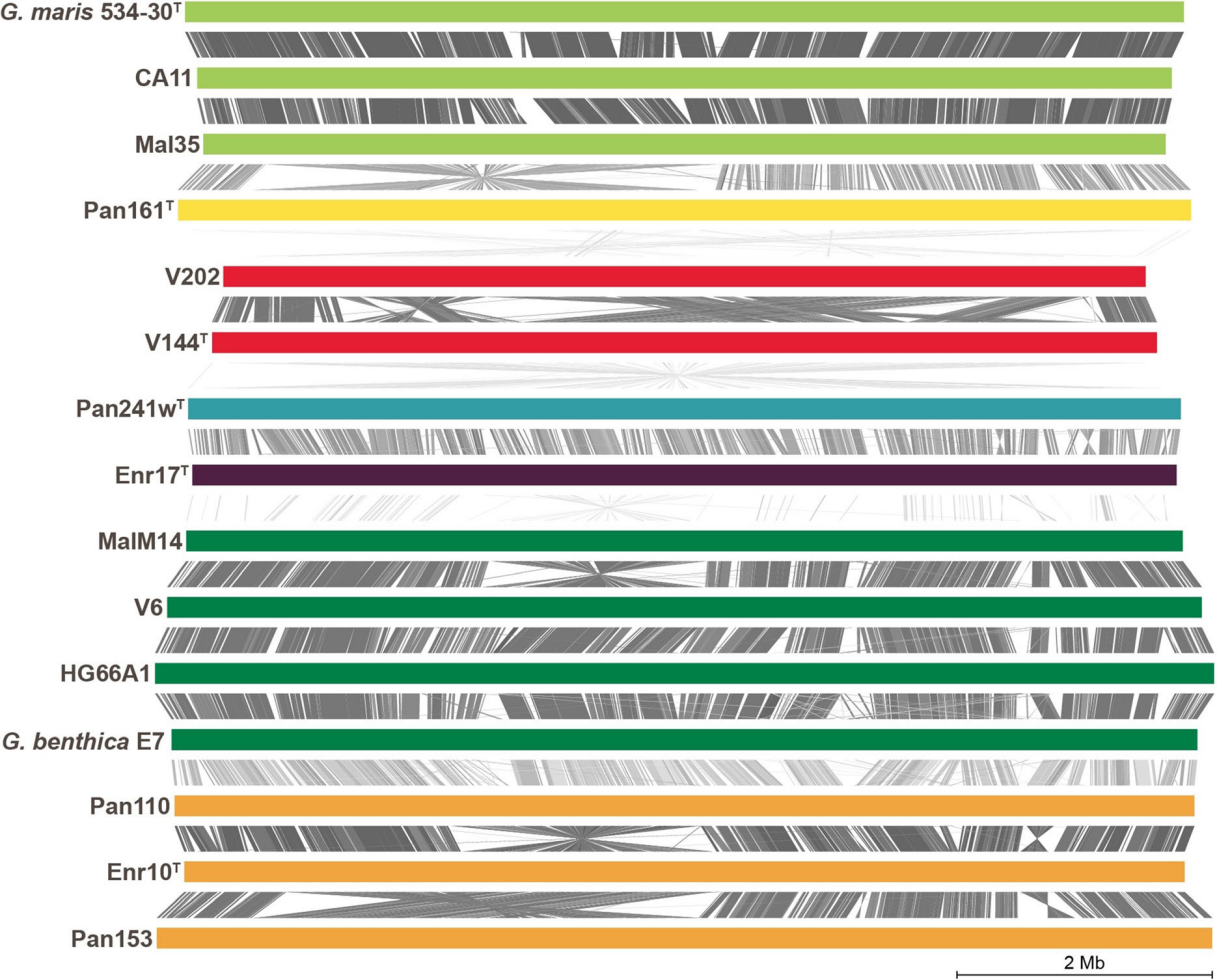
Nucleotide-based local alignment of complete genomes. Each genome is represented by a coloured bar corresponding to its size and group affiliation ((I) light green, (II) yellow, (III) red, (IV) blue, (V) purple, (VI) dark green and (VII) orange). Identity values between 73% (light grey) and 100% (dark grey) are given for homologous sections. *G. chilikensis* was not included in the analysis as its genome is not closed. (Color figure online)

When locally aligning these new genomes, the phylogeny-based group affiliations become clearly visible (Fig. 6). Genomes within a group share high identity values for long sections, sometimes interrupted by inversions of complete sections. Genomes of different groups only share fewer sections with lower identity values.

**Fig. 7:**
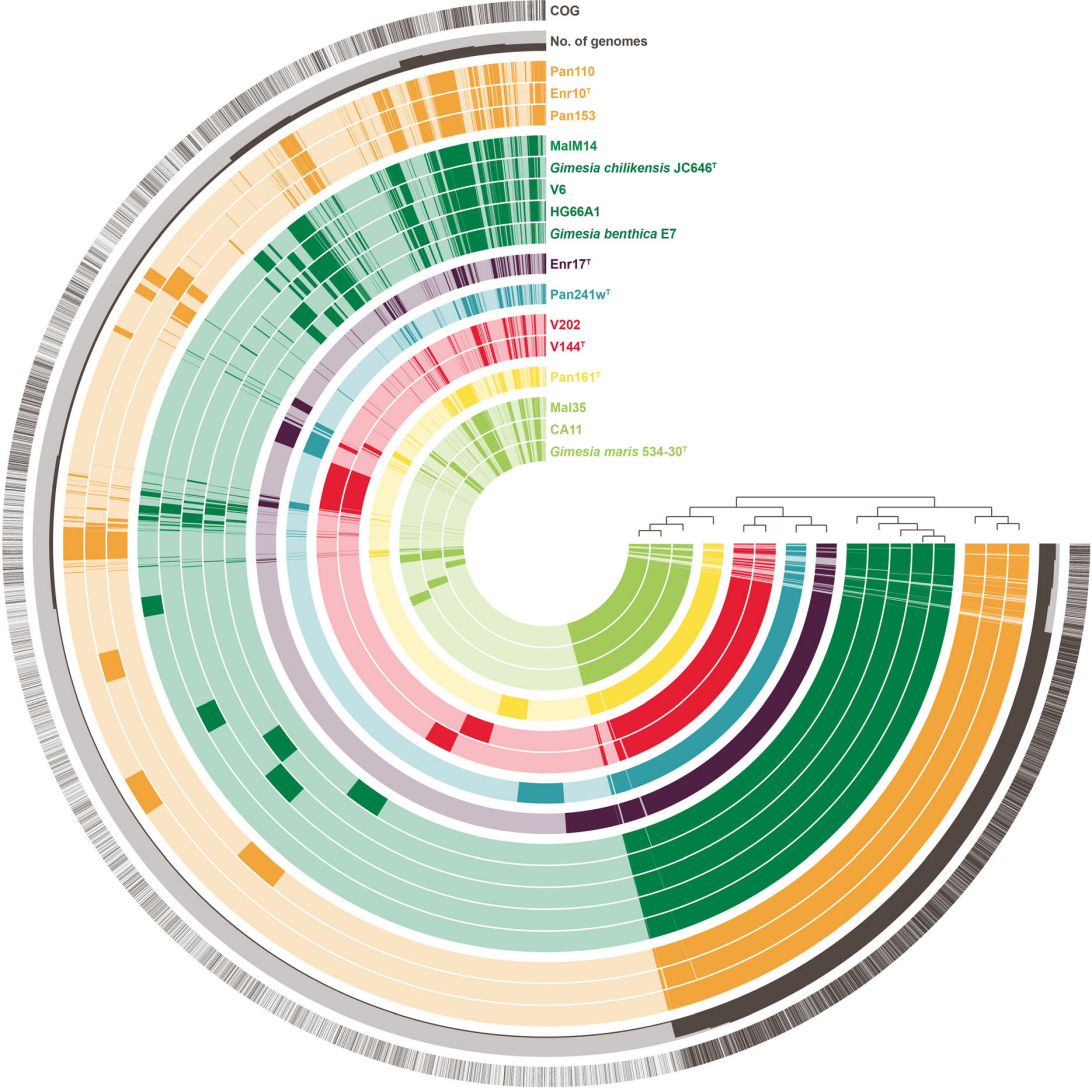
Pan genome of all strains described here. Each open circle represents the pan genome of all strains but is coloured darker when the gene is present in the respective genome. Colours represent the group affiliation of the genomes: (I) light green, (II) yellow, (III) red, (IV) blue, (V) purple, (VI) dark green and (VII) orange. The trees reflect on the relatedness of the strains based on the absence/presence of genes. The outer circles show the number of genomes in which a gene is present and if it has a COG annotation (dark grey). (Color figure online)

The determined groups are also confirmed by the analysis of their pan genome (Fig. 7). In addition to genes present in all genomes (3–5 o’clock position, Fig. 7), genes present in most genomes (5–6 o’clock, Fig. 7) and genes that are only present in one genome (6–10 o’clock, Fig. 7), there are also genes only found in members of one group (10–12 o’clock, Fig. 7), thereby defining the respective groups.

Taken together, based on the phylogenetic analysis, the alignment of the genomes, the absence and presence of gene clusters as well as morphological and physiological data, we propose that each group (I to VII) corresponds to one species within the genus *Gimesia*. We propose the names *Gimesia algae* for strain Pan161^T^ (group II), *Gimesia aquarii* for strains V202 and V144^T^ (group III), *Gimesia alba* for strain Pan241w^T^ (group IV), *Gimesia fumaroli* for strain Enr17^T^ (group V), and *Gimesia panareensis* for strains Enr10^T^, Pan110 and Pan153 (group VII), whilst we conclude that strains CA11 (= DSM 101991 = LMG 29074) and Mal35 (= LMG 31348 = CECT 9838 = VKM B-3428) are novel isolates of *G. maris* (group I) and strains V6, HG66A1 and MalM14 are novel isolates of *G. chilikensis* (group VI).

### Emended description of the genus *Gimesia* (Scheuner et al. 2014) Kumar et al. 2020

The description is the one given previously (Kumar et al. 2020), with the following modifications. Major fatty acids are palmitic acid (16:0) and a fatty acid with the equivalent chain length of 15.817. The G+C content of the genomic DNA is between 45.1 and 53.7%.

### Emended description of *Gimesia chilikensis* Kumar et al. 2020

The description is the one given previously (Kumar et al. 2020), with the following modifications. Cell sizes are 1.2–1.9 × 0.7–1.0 µm. Most cells are at least temporarily flagellated and motile. Most cells possess stalks (usually about the length of the cell) opposite of the budding pole. Most cells have crateriform structures covering the entire cell surface. Different strains of the species have temperature optima for growth between 25 and 33 °C.

Additional strains belonging to the species include HG66A1 (= DSM 100825 = LMG 29015; genome acc. no. CP036266, 16S rRNA acc. no. MK554525), V6 (= DSM 29811 = LMG 29078; genome acc. no. CP036347, 16S rRNA acc. no. MK554511) and MalM14 (= CECT 30191 = STH00944, Jena Microbial Resource Collection (JMRC), = LMG 29132; genome acc. no. CP036342, 16S rRNA acc. no. MK554514), isolated from rocky tide land (Helgoland Island, Germany), a seawater aquarium (Braunschweig, Germany) and marine sediment (Mallorca Island, Spain), respectively.

### Description of *Gimesia alba* sp. nov.

*Gimesia alba* (al’ba. L. fem. adj. *alba* white, corresponding to the whitish colour of the cells).

Cells are short grain-rice-shaped (size: 1.4 ± 0.2 × 0.7 ± 0.1 µm) and form rosettes which assemble into aggregates. Cells contain crateriform structures covering the entire cell surface and have a stalk (usually about the length of the cell) opposite of the budding pole. The temperature range for growth is 15–33 °C (optimum 30 °C) and for pH is 6–10 (optimum 7.5). Colonies are white. The genome size of the type strain is 7.77 Mb with a G+C content of 49.6%.

The type strain is Pan241w^T^ (= DSM 100744^T^ = LMG 31345^T^ = CECT 9841^T^ = VKM B-3430^T^), isolated from organic material from the hydrothermal vent system close to Panarea Island, Italy. The type strain genome (acc. no. CP036269) and 16S rRNA gene sequence (acc. no. MK554516) are available from GenBank.

### Description of *Gimesia algae* sp. nov.

*Gimesia algae* (al’gae. L. gen. n. *algae* of an alga, corresponding to the isolation of the type strain from an alga).

Cells are short grain-rice-shaped (size: 1.5 ± 0.3 × 0.7 ± 0.2 µm) and form rosettes which assemble into branched aggregates. Cells contain crateriform structures covering the entire cell surface and have a stalk (usually about the length of the cell) opposite of the budding pole. Grows at 10–30 °C (optimum 26 °C) and at pH 5.5–10.0 (optimum 7.5). Colonies are white. The genome size of the type strain is 7.93 Mb with a G+C content of 50.2%.

The type strain is Pan161^T^ (CECT 30192^T^ =  STH00943^T^, Jena Microbial Resource Collection (JMRC), = LMG 29130^T^), isolated from an alga collected from the hydrothermal vent system close to Panarea Island, Italy. The type strain genome (acc. no. CP036343) and 16S rRNA gene sequence (acc. no. MK554515) are available from GenBank.

### Description of *Gimesia aquarii* sp. nov.

*Gimesia aquarii* (a.qua’ri.i. L. gen. n. *aquarii* of an aquarium, corresponding to the origin of the type strain from an aquarium).

Cells are short grain-rice-shaped (type strain cell size: 1.3 ± 0.2 × 1.0 ± 0.1 µm) and form rosettes which assemble into branched aggregates. Cells have crateriform structures covering the entire cell surface and a stalk (usually about the length of the cell) opposite of the budding pole. The temperature range for growth is 15–30 °C (optimum 27 °C) and pH range is 6.5–9.5 (optimum 8.0). Colonies are orange. The genome size of the type strain is 7.40 Mb with a G+C content of 45.1%.

The type strain is V144^T^ (= DSM 101710^T^ = VKM B-3433^T^), isolated from an ornamental seawater aquarium. The type strain genome (acc. no. CP037920) and 16S rRNA gene sequence (acc. no. MK554556) are available from GenBank. A second strain belonging to the species is V202 (= DSM 104302 = VKM B-3440; genome acc. no. CP037422, 16S rRNA acc. no. MK554536), isolated from the same seawater aquarium as the type strain.

### Description of *Gimesia fumaroli* sp. nov.

*Gimesia fumaroli* (fu.ma.ro’li. L. gen. n. *fumaroli* of a fumarole, corresponding to the provenance of the type strain from a fumarole).

Cells are short grain-rice-shaped (size: 1.9 ± 0.3 × 1.3 ± 0.2 µm) and form rosettes which assemble into loose aggregates. Cells have crateriform structures covering the entire cell surface and a stalk (usually about the length of the cell) opposite of the budding pole. Grows at 10–30 °C (optimum 27 °C) and at pH 6.0–9.5 (optimum 6.5). Colonies are white. The genome size of the type strain is 7.70 Mb with a G+C content of 49.5%.

The type strain is Enr17^T^ (= DSM 100710^T^ = VKM B-3429^T^, synonym Enrichment17), isolated from a marine hot lake gas escape at Panarea island, Italy. The type strain genome (acc. no. CP037452) and 16S rRNA gene sequence (acc. no. MK554524) are available from GenBank.

### Description of *Gimesia panareensis* sp. nov.

*Gimesia panareensis* (pa.na.re.en’sis. N.L. fem. adj. *panareensis* pertaining to Panarea, corresponding to the origin of the type strain from Panarea Island, Italy).

Cells are short grain-rice-shaped (type strain cell size: 1.3 ± 0.1 × 0.8 ± 0.1 µm) and form rosettes which assemble into aggregates. Cells have crateriform structures covering the entire cell surface and a stalk (usually about the length of the cell) opposite of the budding pole. Grows at 15–37 °C (optimum 32 °C) and at pH 5–10 (optimum 7.5). Colonies are cream-coloured. The genome size of the type strain is 7.83 Mb with a G+C content of 53.3%.

The type strain is Enr10^T^ (= DSM 100416^T^ = LMG 29082^T^, synonym Enrichment10), isolated from a rust biofilm at a hot lake gas escape close to Panarea Island, Italy. The type strain genome (acc. no. CP037421) and 16S rRNA gene sequence (acc. no. MK554508) are available from GenBank.

Additional strains belonging to the species are Pan110 (= DSM 100280 = CECT 9839; genome acc. no. CP036277, 16S rRNA acc. no. MK559981) and Pan153 (= DSM 100430 = LMG 29134; genome acc. no. CP036317, 16S rRNA acc. no. MK554531), both isolated from sediments near a newly formed hot lake gas escape at Panarea Island, Italy.

## Electronic supplementary material

Below is the link to the electronic supplementary material.

Supplementary material 1 (DOCX 872 kb)

Supplementary material 2 (XLSX 10 kb)

Supplementary material 3 (XLSX 29 kb)

Supplementary material 4 (XLSX 10 kb)

Supplementary material 5 (XLSX 19 kb)
