# Supplementary material for: Additions to the genus Gimesia: description of Gimesia alba sp. nov., Gimesia algae sp. nov., Gimesia aquarii sp. nov., Gimesia aquatilis sp. nov., Gimesia fumaroli sp. nov. and Gimesia panareensis sp. nov., isolated from aquatic habitats of the Northern Hemisphere
Source: Antonie Van Leeuwenhoek. 2020 Nov 24;113(12):1999–2018. doi: 10.1007/s10482-020-01489-0 (PMC7716864; doi:10.1007/s10482-020-01489-0)
Supplement: Supplementary file 1 — Supplementary material 1 (DOCX 872 kb) [file 10482_2020_1489_MOESM1_ESM.docx]

***Supplementary Material***

**Additions to the genus *Gimesia*: Description of *Gimesia alba* sp. nov., *Gimesia* *algae* sp. nov., *Gimesia aquarii* sp. nov., *Gimesia aquatilis* sp. nov., *Gimesia fumaroli* sp. nov. and *Gimesia panareensis* sp. nov., isolated from aquatic habitats of the Northern Hemisphere**

Sandra Wiegand^1^, Mareike Jogler^2^, Christian Boedeker^3^, Anja Heuer^3^, Patrick Rast^3^, Stijn H. Peeters^4^, Mike S. M. Jetten^4^, Anne-Kristin Kaster^1^, Manfred Rohde^5^, Nicolai Kallscheuer^4^ and Christian Jogler^2,4,^*

^1^Institute for Biological Interfaces 5, Karlsruhe Institute of Technology, Eggenstein-Leopoldshafen, Germany

^2^ Department of Microbial Interactions, Friedrich-Schiller-University, Jena, Germany

^3^ Leibniz Institute DSMZ, Braunschweig, Germany

^4^ Department of Microbiology, Radboud University, Nijmegen, The Netherlands

^5^ Central Facility for Microscopy, Helmholtz Centre for Infection Research, Braunschweig, Germany

* corresponding author: christian.jogler@uni-jena.de

# Supplementary Figures


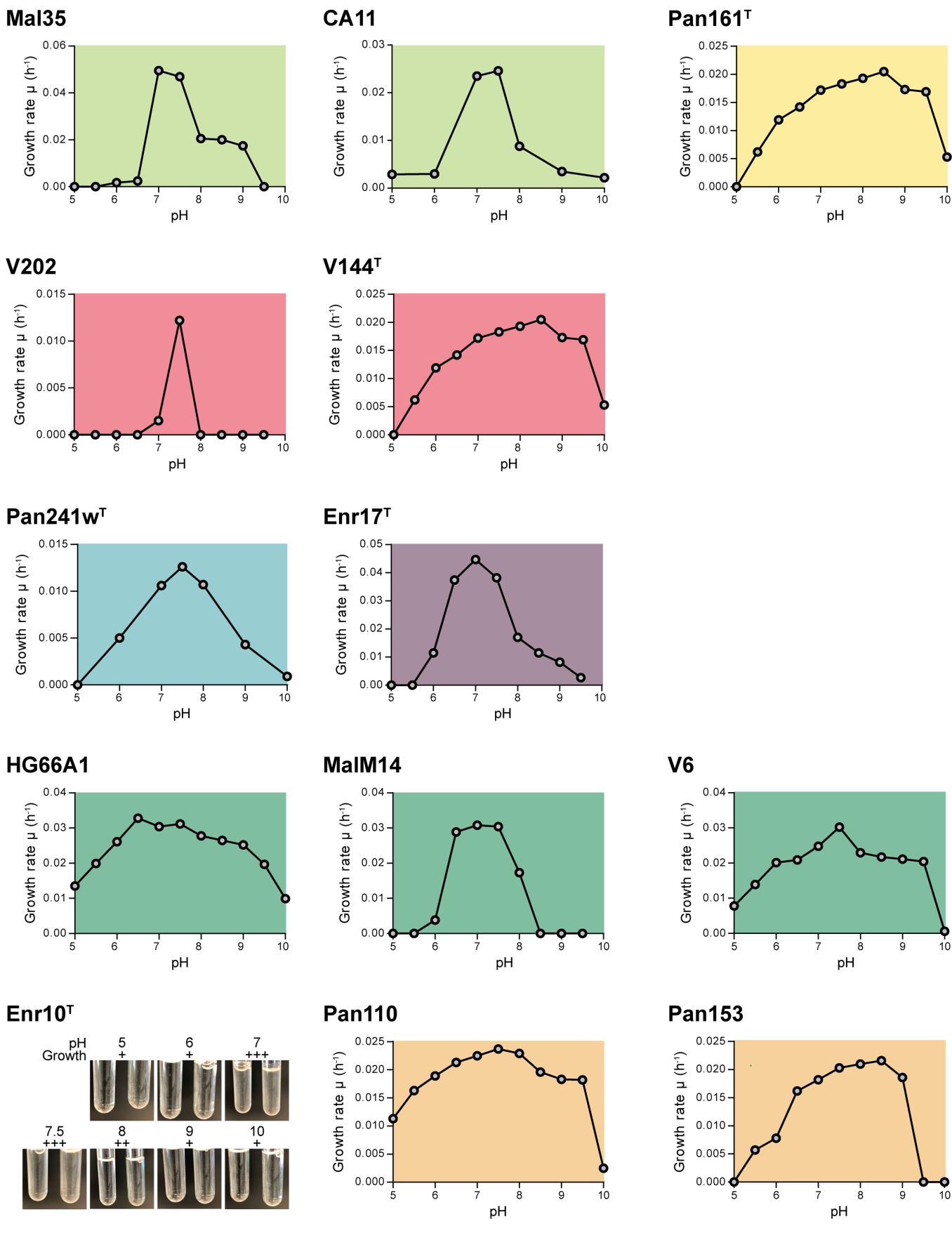


**Figure S1 | Growth at different pH values.** Species suspected to belong to the same species (groups I to VII) have the same colour: (I) light green, (II) yellow, (III) red, (IV) blue, (V) purple, (VI) dark green and (VII) orange. For strain Enr10^T^ the optical density could not be determined due to strong aggregate formation. Hence, no growth rate can be provided for this strain.


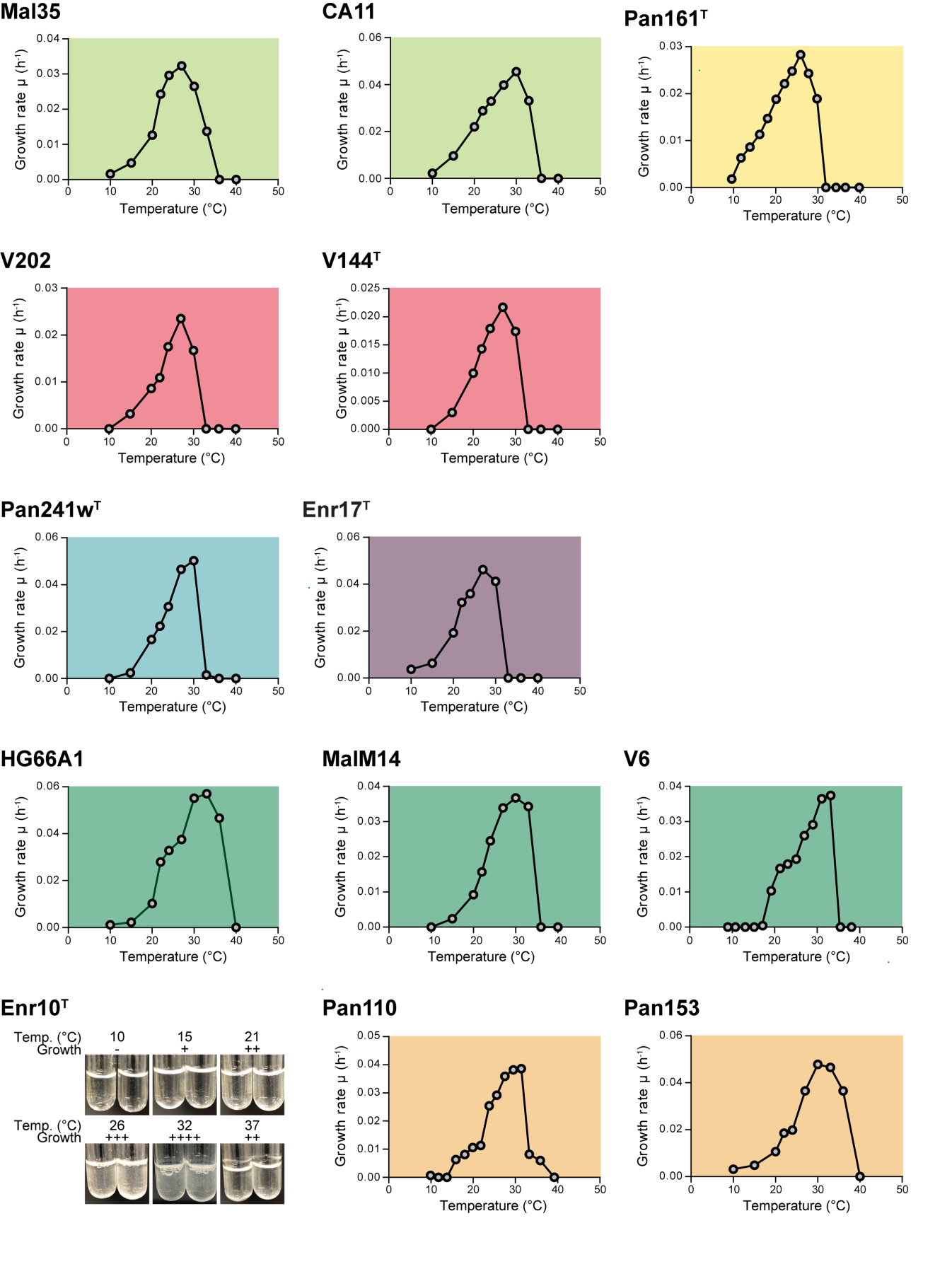


**Figure S2 | Growth at different temperatures.** Species suspected to belong to the same species (groups I to VII) have the same colour: (I) light green, (II) yellow, (III) red, (IV) blue, (V) purple, (VI) dark green and (VII) orange. For strain Enr10^T^ the optical density could not be determined due to strong aggregate formation. Hence, no growth rate can be provided for this strain.

# Supplementary Tables

*Supplementary Tables S1-S4 are provided as separate files.*

**Table S1 |** Sampling and cultivation of all novel strains.

**Table S2 |** Distance marker values determined for 16S rRNA sequence identity, *rpoB* gene identity, average nucleotide identity (ANI), amino acid identity (AAI) and percentage of conserved proteins (POCP). All values are given in percent.

**Table S3 |** Cell sizes of the novel strains.

**Table S4 |** Genome-based analysis of the primary metabolism of all strains. Blue entries indicate enzymes both capable of the same reaction and the different green shades indicate different subunits of the same enzymes.
